# Pilot randomized trial of efficacy and safety of yogic technique versus polyethylene glycol solution for bowel preparation in colonoscopy

**DOI:** 10.1016/j.igie.2024.08.005

**Published:** 2024-08-31

**Authors:** Manas Kumar Panigrahi, Mitali Madhumita Rath, Mohd Imran Chouhan, Rajesh Manik, Ajaya Ghosh R U, Madhav Sameer Makashir, Hemanta Kumar Nayak, Biswa Mohan Padhy, Subash Chandra Samal

**Affiliations:** 1 Department of Gastroenterology, All India Institute of Medical Sciences, Bhubaneswar, India; 2 Department of Pathology, IMS & SUM-II Medical College and Hospital, Phulnakhara, Bhubaneswar, India; 3 Department of Medicine, Government Medical College, Rajouri, Jammu and Kashmir, India; 4 Department of Yoga, Kalinga University, Nava Raipur, Chhattisgarh, India; 5 Department of Pharmacology, All India Institute of Medical Sciences, Bhubaneswar, India

## Abstract

**Background and Aims:**

The traditional yogic technique of shankha prakshalana (SP) has been known to cleanse the bowel, but its efficacy as a bowel preparation agent in colonoscopy has not been studied widely. We compared the efficacy and safety of SP with split-dose polyethylene glycol (PG) in bowel preparation for colonoscopy.

**Methods:**

Patients undergoing elective colonoscopy were randomized into 2 groups: PG group (n = 47) and SP group (n = 47). Patients in the PG group were given 2 liters of PG 3350 in a split-dose regimen. Patients in the SP group were administered 400 mL of lukewarm saline water followed by a set of 5 asanas (physical exercises) of SP, with each asana performed 8 times under the supervision of a certified yoga trainer (R.M.). This constituted 1 cycle, which was repeated 6 times (total intake of 2400 mL lukewarm saline water). The Boston Bowel Preparation Scale (BBPS) was used to assess the main outcome of the efficacy of bowel preparation.

**Results:**

A total of 94 patients were enrolled, with 47 in each study group. The mean BBPS for the whole colon in the SP group (8.15 ± 1.02) was higher than that in the PG group (7.55 ± 1.08; *P* = .007). Moreover, the segmental BBPS for the right side of the colon was significantly higher in the SP group (2.64 ± 0.48) than in the PG group (2.36 ± 0.60; *P* = .010). Adverse effects, such as nausea (31.9% vs 4.3%; *P* = .001), bloating (27.7% vs none; *P* < .0001), and disturbed sleep (63.8% vs 12.8%; *P* < .0001), were significantly more frequent in the PG group than in the SP group.

**Conclusions:**

The traditional yogic technique of shankha prakshalana is an effective method for bowel preparation in colonoscopy, with overall better efficacy and safety, compared with the standard split-dose PG regimen. (Clinical trial registration number: CTRI/2020/07/026899.)

**Highlights**

**What is already known?**

• Polyethylene glycol (PG) electrolyte solution is the usual method of bowel preparation for colonoscopy.
• Acceptability, unpalatable taste, and affordability are some of the issues related to the PG electrolyte solution.
• Shankha prakshalana is a traditional yogic technique known for its laxative use but has not been widely studied as a method of bowel preparation for colonoscopy.

**What is new in this study?**

• The traditional yogic technique of shankha prakshalana is an effective, acceptable, affordable, and relatively safe method of bowel preparation for colonoscopy.
• Shankha prakshalana is overall better as a bowel preparation method for colonoscopy with fewer adverse events compared with standard PG electrolyte solution.

Colonoscopy is the criterion standard test for the evaluation of colonic mucosa.^1^ It requires adequate bowel preparation for proper mucosal examination to detect and diagnose colorectal lesions.^2^ Polyethylene glycol (PG) electrolyte solution is one of the most commonly used methods for colon preparation.^3^ The American Society for Gastrointestinal Endoscopy 2014 guidelines recommend the use of a split-dose bowel cleansing regimen for elective colonoscopy.^4^

In the split-dose preparation method, it is recommended that the standard dose of a bowel preparation is split between the day before and the morning of the procedure.^4^ This often leads to disturbed sleep and inconvenience while traveling to the hospital. In addition, the volume of solution to be ingested and its disagreeable taste may prove to be hindrances in patient acceptability. Although the split-dose bowel preparation is widely followed, these issues could negatively affect its clinical application.

Shankha prakshalana (SP), a traditional yogic bowel-cleansing technique, simply involves drinking saline water followed by 5 yogic postures (asanas).^5^ It is a natural method that has fewer adverse effects and better tolerability. It can be performed within a few hours before the planned procedure, thereby causing less inconvenience to the patient. The role of SP has been studied for hypertension^6^ and chronic low back pain^7^ and has been found to be effective and safe in these studies. However, SP as a bowel preparation method has not been studied widely.

Therefore, we designed the present study to compare the efficacy and safety of SP with split-dose PG preparation in bowel cleansing for colonoscopy.

## Methods

### Study design

This was a prospective, single-center, randomized, single-blinded (the endoscopist was blinded), parallel-group, active-control trial conducted in the Department of Gastroenterology, All India Institute of Medical Sciences, Bhubaneswar, Odisha, India during the period of August 2020 to February 2021.

The study was registered with the Clinical Trials Registry—India (CTRI/2020/07/026899) and reported according to the Consolidated Standards of Reporting Trials guidelines for randomized controlled trials. Approval of the study was obtained from the institutional ethical committee, ref no. T/IM NF/Gastro/19/77.

### Participants

Study participants included adult patients (age ≥18 years) of either sex scheduled to undergo a medically indicated elective colonoscopy. Patients with comorbid conditions (diabetes mellitus, hypertension, chronic liver disease, acute or chronic heart failure, arrhythmic disorder, renal failure, dyselectrolytemia) or suspected or previously diagnosed intestinal stricture of any etiology, pregnant or lactating women, patients on anticoagulants, patients with history of any abdominal surgery, psychiatric disease limiting the ability to complete the preparation, altered consciousness, chronic neurologic diseases, use of opiates/narcotics, antidiarrheal medications or oral iron supplementation in the last 14 days before colonoscopy, chronic constipation as defined by ROME-III criteria, or history of an allergic reaction to PG, patients unable or unwilling to consume PG solution or unable to complete all the asanas of SP, patients with history of inadequate bowel preparation, and patients refusing to participate or unable to provide informed consent were excluded from the study.

### Randomization and blinding

Enrolled patients were randomized into 2 groups in a 1:1 ratio by means of a computer-generated random number table. The numbers were assigned consecutively by a nurse (A.G.R.U.), who was blinded to the allocation, when the appointment for colonoscopy was given. The endoscopist (H.K.N.) collecting data for outcome assessment was blinded to participant allocation. Participants were told not to disclose their study allocation to the endoscopist or investigators. Allocation concealment was carried out with opaque sealed envelopes.

Written informed consent was obtained from every participant. The trial was conducted in accordance to good clinical practices and the Declaration of Helsinki.^8^

### Bowel preparation techniques/regimens

Both study groups were advised to take a low-residue diet (total fiber less than 10 g/d) 1 day before the day of the colonoscopy. The patients were not permitted to use laxatives, enema agents, or bowel-cleansing agents other than those used on the days leading to the study.

#### PG group

PG electrolyte powder was diluted in 2 liters of water, according to the manufacturer’s instructions. After dilution, 2 liters of the solution contained 18 mEq/L PG, 125 mEq/L sodium, 10 mEq/L potassium, 35 mEq/L chloride, 80 mEq/L sulphate, and 20 mEq/L bicarbonate. The standard split-dosing method was used. One half of the PG solution (ie, 1 L) was given the night before and the other half (ie, another 1 L) was given in the morning on the day of the colonoscopy. The morning dose was administered 3 to 8 hours before the procedure. Participants were advised to drink water after completion of the 2-L PG solution until they passed watery stools.

#### SP group

**Figure 1:**
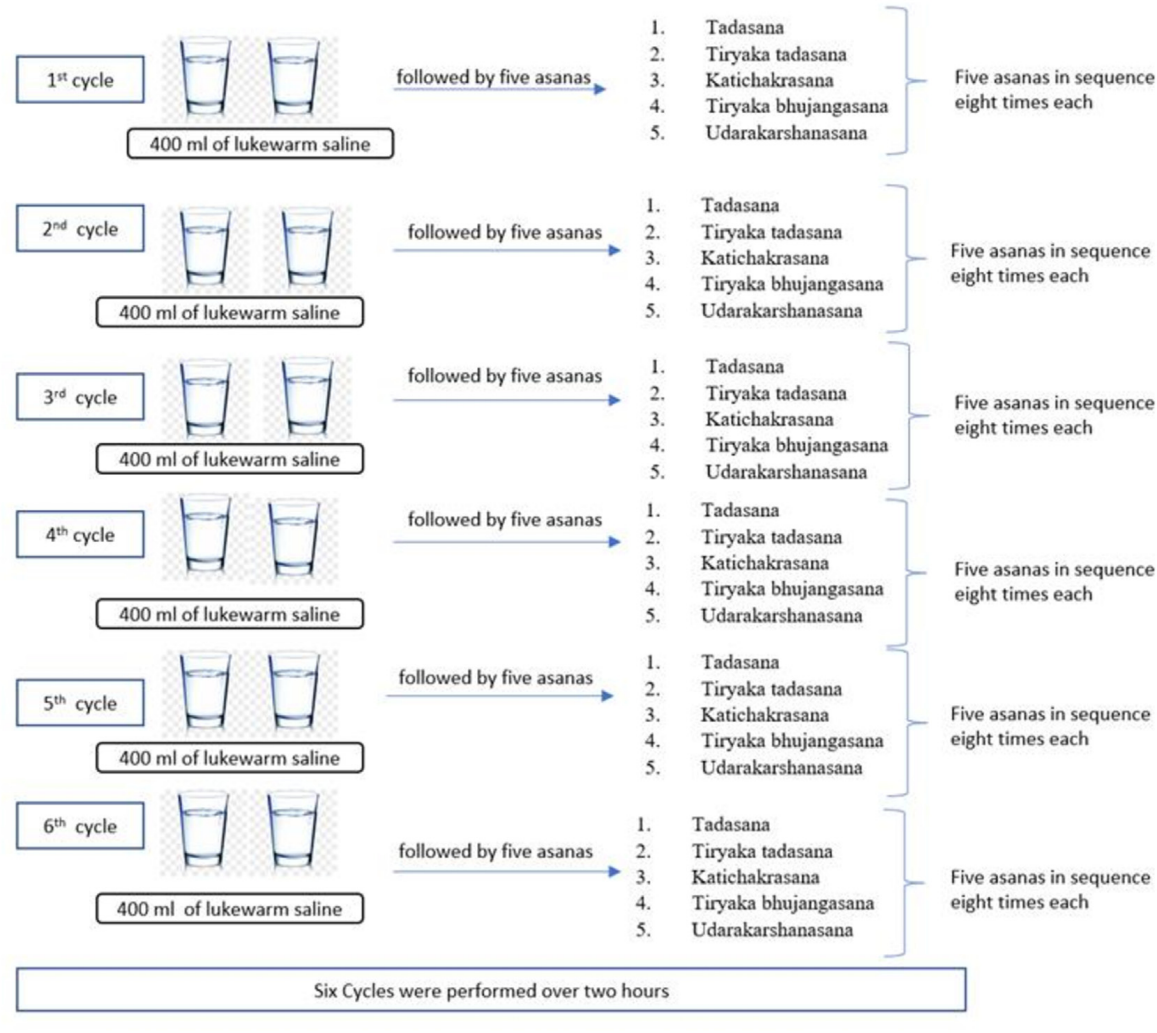
Shankha prakshalana.

The SP technique was administered under the supervision of a yoga trainer (R.M.) in the morning before the colonoscopy. Lukewarm saline water solution was prepared by dissolving 9 grams (2 teaspoons) of salt in 1 liter of lukewarm water. Patients were administered 400 mL of this lukewarm saline water followed by 5 asanas (yogic postures) of SP sequentially, with each asana performed 8 times (Fig. 1). This constituted 1 cycle. A total of 6 such cycles were performed. At the beginning of each cycle, the patient was advised to drink 400 mL of lukewarm saline water. Patients were instructed to interrupt the process whenever they had the urge to defecate. Regardless of the bowel movement, they were instructed to complete the 6 cycles of asanas. The SP regimen was completed within 1.5 to 2 hours. All colonoscopy examinations were performed within 2 hours of patients’ completion of the SP regimen.

**Supplementary Figure 1:**
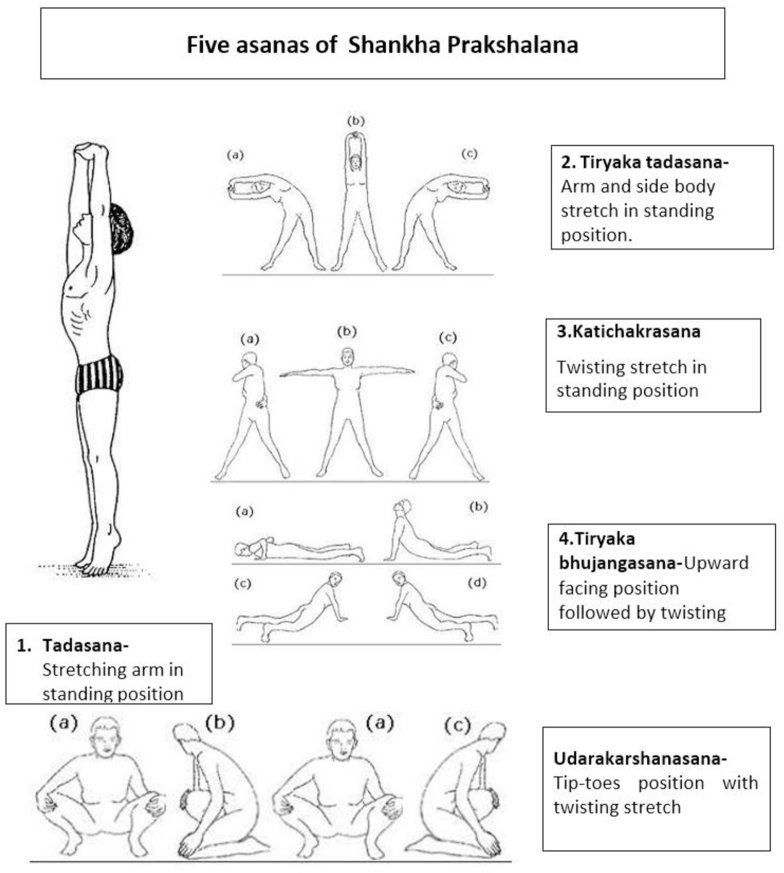


Five asanas were performed in the following sequence (Appendix 1 and Supplementary Fig. 1, available online at www.igiejournal.org):

1. Tadasana (stretching arm in standing position)
2. Tiryaka tadasana (arm and side body stretch in standing position)
3. Katichakrasana (twisting stretches in standing position)
4. Tiryaka bhujangasana (upward facing dog position followed by a twisting stretch)
5. Udarakarshanasana (tiptoes position with twisting stretch)

### Endoscopic procedure

An Olympus (Tokyo, Japan) CF-H170L endoscope was used for performing the colonoscopies. All the endoscopies were done by a single experienced endoscopist (H.K.N.). Any unpleasant adverse effects, including nausea, vomiting, pain in the abdomen, bloating, chest pain, dizziness, or disturbed sleep, were noted.

### Outcomes and efficacy assessment

The primary outcome was the adequacy of bowel preparation in colonoscopy evaluated according to the Boston Bowel Preparation Scale (BBPS). The secondary outcomes included adverse events related to bowel preparation in colonoscopy and willingness to repeat the procedure in the future.

The efficacy of colon cleansing was assessed with the use of the BBPS (Supplementary Table 1, available online at www.igiejournal.org).^9^ Each segment of the colon (left, transverse, and right) was given a score ranging from 0 to 3 by the endoscopist based on the bowel preparation adequacy. The final score was calculated by summing the score from the 3 segments, with a maximum total score of 9. If the procedure had to be aborted owing to poor bowel preparation according to the endoscopist’s discretion, then a score of 0 was given to each bowel segment that could not be examined.

After completion of the procedure, participants in the SP group who had undergone colonoscopy in the past using the standard split dose bowel preparation were asked about their preference for bowel preparation between the 2 methods (PG or SP) in case they had to undergo a colonoscopy in future.

### Statistical methods and sample size

SPSS version 22.0 (IBM Corp, Armonk, NY, USA) was used to analyze the data. Quantitative data were expressed as mean ± standard deviation (SD), and categoric data were expressed as n (%). Descriptive statistics for measured variables were expressed as range, mean, and SD (for metric data). Outcomes of both groups were compared with the use of the Fischer exact test or chi-square test for categoric measures and by independent-samples *t* test for quantitative data. A *P* value <.05 was considered to be significant.

Assuming that SP would be at least 15% superior to PG, using the previously reported mean BBPS with PG of 6.88 ± 1.78 and threshold score 6, the total sample size of 94, with 47 in each group was calculated to provide 5% significance and 80% power.

## Results

**Figure 2:**
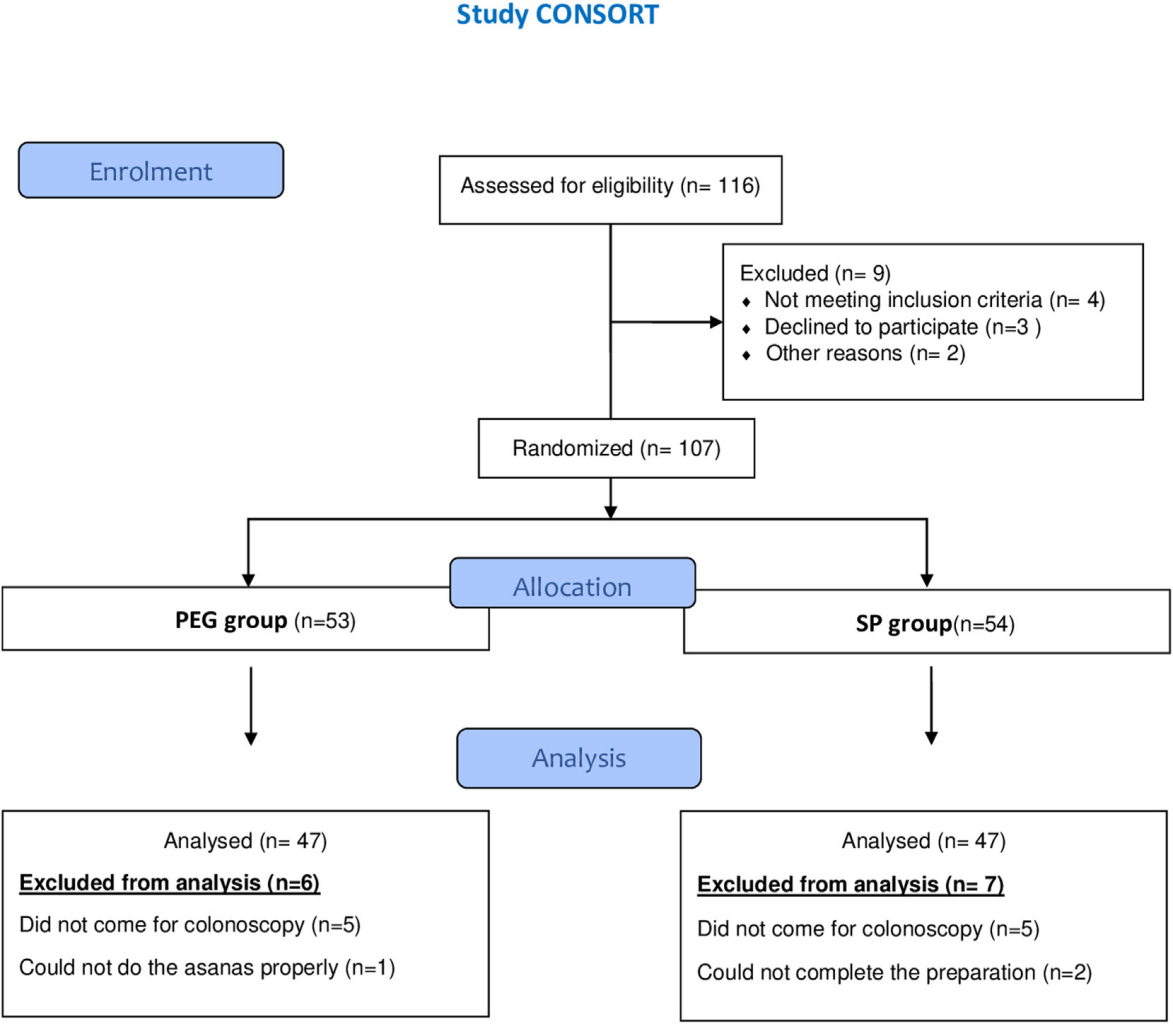
Study CONSORT diagram. *PG*, Polyethylene glycol; *SP*, shankha prakshalana.

A total of 116 patients who presented to our outpatient department and required colonoscopy were screened for eligibility. Among these, 107 patients fulfilled the inclusion criteria and were randomized into the 2 groups, 54 in the PG group and 53 in the SP group. Ten patients did not show up for the procedure (5 in the PG group and 5 in the SP group), and 3 patients could not complete the prescribed colonoscopy preparation method (2 in the PG group and 1 in the SP group) and were also excluded from the study (Fig. 2).

The mean ages in both groups (PG vs SP) were similar (44.68 ± 13.64 years vs 39.66 ± 12.47 years; *P* = .066). Men constituted 70.21% (n = 66) of the participants. Overall, indications for colonoscopy between the 2 groups also were similar (Table 1).

**Table 1:** Baseline characteristics

	PG (n = 47)	SP (n = 47)	*P* value
Age, y	44.68 ± 13.64	39.66 ± 12.47	.066
Male sex	31 (65.96)	35 (74.47)	.499
BMI, kg/m^2^	22.24 ± 1.05	22.16 ± 0.98	.783
Indications			
Abnormal radiologic findings	13	11	
Change in bowel habits	10	12	
Screening	4	5	
Polyp surveillance	4	6	
Abdominal pain	5	3	
Anemia	6	7	
IBD (follow-up)	3	2	
Others	2	1	
Median time from start of regimen to start of colonoscopy, min	1020 (900-1200)	145 (120-240)	<.001

Values are presented as mean ± standard deviation, n (%), n, or median (interquartile range).

*BMI*, Body mass index; *IBD*, inflammatory bowel disease; *PG*, polyethylene glycol; *SP*, shankha prakshalana.

### Adequacy of bowel preparation

The mean BBPS for the whole colon and segmental colon were better in the SP group than in the PG group (8.15 ± 1.201 vs 7.55 ± 1.080, respectively, for whole colon; *P* < .05), although the 15% margin of superiority was not achieved (Table 2). In the PG group, 1 patient had BBPS ≤6, whereas in the SP group, 100% had adequate bowel preparation.

**Table 2:** Bowel preparation quality (BBPS)

Colonic segment	PG group score	SP group score (n = 47)	*P* value	Difference	95% CI of difference
Whole colon	7.55 ± 1.080	8.15 ± 1.201	.007	+0.6 ± 0.217	+0.16 to +1.10
Left side of the colon	2.53 ± 0.54	2.65 ± 0.478	.231	+0.12 ± 0.105	−0.08 to +0.33
Transverse colon	2.66 ± 0.479	2.80 ± 0.397	.104	+0.14 ± 0.908	−0.03 to +0.32
Right side of the colon	2.36 ± 0.605	2.64 ± 0.485	.016	+0.27 ± 0.113	+0.05 to +0.50

Values are presented as mean ± standard deviation.

*BBPS*, Boston Bowel Preparation Scale; *CI*, confidence interval; *PG*, polyethylene glycol; *SP*, shankha prakshalana.

In the SP group, detected lesions included 2 colonic polyps, 6 terminal ileal erosions/ulcers, 3 colonic ulcers, 4 diverticula, 1 rectal growth, and 11 hemorrhoids. In the PG group, detected lesions included 3 colonic polyps, 8 terminal ileal erosions/ulcers, 5 colonic ulcers, 2 diverticula, 12 hemorrhoids, and 1 worm infestation.

### Frequency of adverse events

Adverse events were significantly more frequent in the PG group than in the SP group (Table 3). The adverse events observed were nausea, vomiting, abdominal pain, bloating, dizziness, and disturbed sleep. The frequencies of nausea, bloating, and disturbed sleep were significantly higher in the PG group compared with the SP group (nausea: 31.9% in the PG group vs 4.3% in the SP group [*P* = .001]; bloating: 27.7% in the PG group vs 0% in the SP group [*P* < .0001]; disturbed sleep: 63.8% in the PG group vs 12.8% in the SP group [*P* < .0001]).

**Table 3:** Adverse events

	PG (n = 47)	SP (n = 47)	*P* value
Nausea	15 (31.9%)	2 (4.3%)	.001
Vomiting	4 (8.5%)	1 (2.1%)	.363
Abdominal pain	6 (12.8%)	1 (2.1%)	.111
Bloating	13 (27.7%)	0 (0%)	<.0001
Dizziness	3 (6.4%)	1 (2.1%)	.617
Disturbed sleep	30 (63.8%)	6 (12.8%)	<.0001

*PG*, Polyethylene glycol; *SP*, shankha prakshalana.

There were 6 participants in the SP group who had undergone colonoscopy in the past using standard split-dose bowel preparation. All of them showed a high willingness to repeat the SP method in the future, in case they needed any further colonoscopy.

## Discussion

In the present study, patients who underwent the SP method of bowel preparation had a higher mean BBPS for the whole colon than those who used the PG-electrolyte solution (8.15 and 7.55, respectively; *P* = .007). In addition, the SP group demonstrated a higher mean BBPS in the right side of the colon compared with the PG group (2.64 and 2.36, respectively; *P* = .01), showing that the SP regimen may be effective in cleansing the right side of the colon as well. This is in contrast to the generally accepted fact that the quality of preparation is poorer in the right side of the colon than in the left side of the colon.^10^

The results of the present study suggest that the ancient technique of SP, using saline water and yoga, is an effective method of bowel preparation for colonoscopy. Moreover, it is certainly safe and well tolerated and has better acceptance among patients.

Yoga originated in ancient times. It refers to the union of mind and body achieved through a system of physical, spiritual, and moral directives. “Shankha” means “conch,” and “prakshalana” means “to clean,” which means cleansing the entire gastrointestinal tract.^7^ The lukewarm saline water given to the patient in the SP method helps in retaining water in the intestinal lumen and lubricating the stool, allowing easy passage. It is postulated that each yoga asana leads to a precise physiologic action in the body. Tadasana opens the pyloric sphincter. Tiryaka tadasana contracts the small intestine. Katichakrasana twists the intestine, which helps in the distal passage of water and stool. Tiryaka bhujangasana opens the ileocecal valve. Udarakarshanasana causes alternate contraction and relaxation of the large intestine and finally evacuation of feces.^11^ Theoretically, a bolus intake of fluid should lead to a greater amount of gastric emptying compared with intermittent drinking. Moreover, saline load is known to stimulate the gastrocolic reflex.^11^ However, further exploration of the exact physiologic mechanisms of this traditional practice may be needed.

To our knowledge, only 2 previous studies^10^^,^^12^ have evaluated yogic methods for bowel preparation. In a nonrandomized, nonblinded pilot study with a small sample size, the authors compared a yogic method with single-dose PG regimen and found that 88.9% of participants in the yoga group had excellent or optimum bowel preparation compared with 77.8% in the PG group. In that study, BBPS, which is a validated and widely accepted score for bowel preparation, was not used; instead, a 4-point scale in 6 sites of the colon was used to assess the quality of bowel preparation.^12^ In another randomized single-center study, lukewarm saline water combined with sequential posture changes (termed by the authors as “shudh colon cleanse”) was compared with a combination single-dose PG-based solution and bisacodyl tablets. The authors found the former to be noninferior to the latter regarding bowel preparation quality, with the advantage of a shorter bowel preparation time.^12^ SP has never been systematically compared with split-dose PG. Recently, a retrospective observational study by Panigrahi et al^13^ evaluated the role of SP in bowel preparation for colonoscopy. However, it was a nonrandomized study and therefore randomized trials on this simple, yet innovative, method of bowel preparation for colonoscopy are needed.

Split-dose PG method is a standard method of colon preparation and has been found to be one of the best methods of colon cleansing in multiple studies.^14^^,^^15^ Many studies have shown the split-dose PG method to have better tolerability and patient acceptance than single-dose lavage and other methods of colon preparation.^16^ In the present study, participants had better tolerability and acceptance of SP in comparison to split-dose PG. A recent study that compared sleep disturbance, bowel movement kinetics, and travel disruption among various bowel preparation regimens has shown that split-dose preparations lead to greatest sleep disturbance. Although there were no differences between various regimens in travel disruption, more frequent disruptions were seen in patients traveling for more than 1 hour.^17^ In practice, patients using split-dose preparation for colonoscopies scheduled in the morning have to wake up in the early morning to take the second dose, causing inconvenience. Because our study was an outpatient study, patients coming for colonoscopy after taking the morning dose had difficulty while traveling. Participants in the SP group required only 2 hours to complete the bowel preparation and did not require any preparation before reaching the hospital or before traveling, causing less inconvenience.

Previous studies have shown fewer adverse effects with the split-dose PG method compared with single-dose lavage and other methods of colon preparation. They have also demonstrated that split-dose preparations lead to decreased intensity of bowel movements, less patient inconvenience, and increased polyp detection rates compared with single-dose regimens.^18^^,^^19^ Our study convincingly demonstrates that adverse effects were significantly less frequent in the SP group than in the split-dose PG group. Nausea, bloating, and disturbed sleep were significantly less frequent in the SP group than in the PG group. Other adverse effects, such as vomiting, abdominal pain, and dizziness were similar in frequency between the 2 groups. Although our study did not include renal parameters as an outcome, it has been shown previously that lukewarm saline water combined with posture changes has no significant long-term effect on renal function or electrolyte levels compared with PG.^20^

The optimal preparation for outpatient colonoscopy continues to be an important area of interest and study. The SP method has many attributes of an ideal bowel preparation method as recommended by the American Society for Gastrointestinal Endoscopy.^21^ SP is inexpensive, reliably empties the colon in a rapid fashion without causing any gross alteration of the colonic mucosa, and has good patient acceptability. Although the precise mechanism of action remains to be explored, the present study demonstrates sufficient levels of efficacy for SP to warrant further exploration of this method. Thus, SP regimen may offer a viable alternative for many patients.

This trial had certain limitations. It was conducted on outpatients, and the results may not be generalizable to hospitalized patients. In addition, patients with limitations to physical activity may not be able to perform SP. Patients in the SP group were mainly young to middle-aged, with a mean age of 39.66 ± 12.47 years. Although allocation concealment was done, it was not adjusted, which might affect the mean age of both groups. Whether the benefits of SP can be generalized to the elderly age group remains to be seen. Furthermore, the exact amount of fluid in the PG group could not be calculated, because patients drank water liberally after the completion of the split-dose preparation. We did not evaluate the changes in serum electrolytes after the procedure, because we did not expect major electrolyte disturbances. A previous study with similar methodology showed that although there were slight changes in serum electrolytes, none of the patients had any clinical abnormalities.^10^ We did not measure preprocedure and postprocedure blood pressure of the patients. However, administering saline water is not likely to cause changes in blood pressure because the patients lose a significant amount of fluid through the intestine. Because this was a pilot study and the first of its kind, we tried to evaluate the effectiveness and safety of SP for bowel preparation before colonoscopy. We have not looked into the total procedure time, withdrawal time, total laxative drug intake and water dose, and adenoma detection rate. It will be better if the BBPS evaluation is done by a neutral endoscopist, which was not done in this study. There are potential issues with generalizing this technique to a broader, more diverse patient population. This was a single-center study with small sample size, so adequately powered multicenter studies with larger sample size would be needed in the future for subgroup analyses.

## Conclusion

In this randomized trial, the traditional yogic technique of shankha prakshalana showed an overall better bowel preparation than split-dose PG. This is a positive study, where the mean BBPS in the SP arm was higher than that in the PG arm. However, the superiority margin of 15% was not achieved, ie, SP is not superior to PG. SP had better tolerability, acceptability, and fewer adverse effects. Thus, SP is a simple, innovative, and effective method of bowel preparation for colonoscopy. Future multicenter studies on different population groups are warranted to establish its generalizability. Therapeutic potential of this technique also needs to be explored in future.

## Disclosure

All authors disclosed no financial relationships.

## Appendix 1

The following sequence of asanas completes 1 cycle of the sankha prakshalana technique. For each asana, the patient is advised to follow the steps listed.

### 1. Tadasana (stretching arm in standing position)

1. Stand in an erect position with gaze fixed at a point directly above and in front with feet 10 to 12 inches apart.
2. While inhaling, raise the arms with fingers of both hands kept interlocked with the simultaneous raising of heels and stretching the whole body upward as far as possible, keeping the hands interlocked and arms straight.
3. Maintain this position for a second.
4. Then turn the palm downward and lower the heels and arm together while exhaling. Once heels touch the ground, rest the hands over the head.

### 2. Tiryaka tadasana (arm and side body stretch in standing position)

1. Stand in an erect position with a fixing gaze at a point directly above and in front with feet 10 to 12 inches apart.
2. While inhaling, raise the arms with fingers of both hands kept interlocked with the simultaneous raising of heels and stretching the whole body upward as far as possible, keeping the hands interlocked and arms straight.
3. With this position maintained and while inhaling, body is to be bent to the right with both arms pressing the ears. Throughout the stretch movement, the patient will be advised to stay on tiptoes.
4. Bring body back to center while exhaling, followed by bending to the left while exhaling, keeping arms parallel to the trunk and pressed against the ear. Bring the body back to center while exhaling.

### 3. Katichakrasana (twisting stretches in standing position)

1. Stand in an erect position with feet kept 10 to 12 inches apart.
2. Place the right hand over the left shoulder and then the left arm wrapped behind the back, and with simultaneous inhaling, twist the body to the left while looking down over the left shoulder and beyond the heels. Keep legs straight and feet firmly placed on the ground while twisting. For a few seconds, keep body in this final twisted position.
3. Bring the body back to the center while exhaling.
4. Place the left hand over the right shoulder then the right arm wrapped behind the back, and with simultaneous inhaling, twist the body to the right while looking down over the right shoulder and beyond the heels. Keep legs straight and feet firmly placed on the ground while twisting. For a few seconds, keep the body in this final twisted position.
5. Bring the body back to the center while exhaling.

### 4. Tiryaka Bhujangasana (twisting stretches from extended cobra position)

1. Lie down in a prone position and rest for a few seconds.
2. Lift head up while placing palms on floor beside the chest with the fingers in the same line as of shoulder. Lift head and chest up, then stretch elbow to the straight position. Both hands should be vertically straight below the shoulders.
3. With inhalation, twist to the left looking on the left shoulder and gazing to the heels. Maintain right thigh pressed strongly on the ground, keeping the arms straight and back relaxed.
4. Stay in this position for a few seconds with exhalation, then resume the center position.
5. Turn to the right while inhaling and looking on the right shoulder and gazing to the left thigh pressed strongly on the ground, keeping the arms straight and back relaxed. An attempt should be made to keep the navel on the ground.
6. Maintain this position for a few seconds with exhalation, then resume the center position.

### 5. Udarakarshanasana (tiptoe position with twisting stretch)

1. In the squatting position, with feet kept about 8 to 10 inches apart, heels are to be lifted up while hands are placed on knees.
2. Keeping feet fixed to the ground while inhaling, turn to the right side and push left knee to the ground, under the right knee.
3. Then twist head back, looking over the right shoulder while keeping back relaxed.
4. Stay in this position for a few seconds with exhalation, then resume squatting position.
5. With inhalation, repeat this twisting of the body in the opposite direction while looking over the left shoulder and with right knee pushed to the floor.
6. Stay in this position for a few seconds.
7. Return to the starting position while exhaling.
