# Supplementary material for: Pilot randomized trial of efficacy and safety of yogic technique versus polyethylene glycol solution for bowel preparation in colonoscopy
Source: IGIE. 2024 Aug 31;4(1):31–37.e1. doi: 10.1016/j.igie.2024.08.005 (PMC12850760; doi:10.1016/j.igie.2024.08.005)
Supplement: Supplementary Table 1 [file mmc2.docx]

**Supplementary Table 1. The Boston Bowel Preparation Scale**

| Score 0 | Colonic mucosa not visible because of solid stool (not even cleared by water) |
| --- | --- |
| Score 1 | Only a portion of the mucosa is visible in the colonic segment. Rest of the portion not well visible because of opaque liquid, stool residue, or staining. |
| Score 2 | Mucosa of colon seen well in a colonic segment with small fragments of opaque liquid and stool |
| Score 3 | No opaque liquid, stool fragments, or residual staining. Entire colonic mucosa seen well. |
